# Involvement of stanniocalcins in the deregulation of glycaemia in obese mice and type 2 diabetic patients

**DOI:** 10.1111/jcmm.13355

**Published:** 2017-10-09

**Authors:** José Javier López, Isaac Jardín, Carlos Cantonero Chamorro, Manuel Luis Duran, María José Tarancón Rubio, Maria Reyes Panadero, Francisca Jiménez, Rocio Montero, María José González, Manuel Martínez, María Jose Hernández, José María Brull, Antonio Jesús Corbacho, Elena Delgado, María Purificación Granados, Luis Gómez‐Gordo, Juan Antonio Rosado, Pedro Cosme Redondo

**Affiliations:** ^1^ Department of Physiology (Phycell) Veterinary Faculty University of Extremadura Cáceres Spain; ^2^ Animal House of University of Extremadura University of Extremadura Cáceres Spain; ^3^ Manuel Encinas's medical center Extremadura Health Service Cáceres Spain; ^4^ Extremadura County blood bank Mérida Spain; ^5^ Aldea Moret's medical center Extremadura Health Service Cáceres Spain; ^6^ Department of Animal Medicine Veterinary Faculty University of Extremadura Cáceres Spain

**Keywords:** DM2, STC1 and STC2, deregulated glycaemia, glucagon

## Abstract

Stanniocalcins are expressed in the pancreas tissue, and it was suggested a direct correlation between circulating insulin and STC2 concentrations in human. Here, we show a significant correlation between STC1 and both glycaemia and glycosylated haemoglobin among DM2 patients, while DM2 patients who present the greatest glycosylated haemoglobin values exhibited the lowest STC2 expression. However, treatment of patients with antiglycaemic drugs does not significantly modify the expression of both STCs. On the other hand, STC2^‐/‐^ mice that exhibited neonatal and adult overweight further presented deregulated glycaemia when they were feed with a hypercaloric diet (breeding pellet, BP). This alteration is more evident at the early stages of the animal life. Deregulated glycaemia in these mice was confirmed using glucose oral test. In addition, STC2^‐/‐^ mice present enhanced pancreas size; thus, the histological analysis reveals that WT mice respond to BP diet by increasing the size of the pancreatic islets through inducing cell division, and STC2^‐/‐^ mice lack this compensatory mechanism. Contrary, BP fed STC2^‐/‐^ mice show enhanced number of islets but of similar size than those fed with regular pellet. Histopathological analysis demonstrates tissue structure disruption and erythrocytes infiltrations in STC2^‐/‐^ mice, possibly due to the stress evoked by the BP diet. Finally, enhanced glucagon immunostaining was observed in the islet of STC2^‐/‐^ mice, and the glucagon ELISA assay confirmed the increase in the circulating glucagon. Summarizing, we present evidence of the role of STCs, mainly STC2, as a possible early marker during development of diabetes mellitus.

## Introduction

Stanniocalcins (STCs) were initially identified as a fish hormone that regulates calcium and phosphate blood concentration 1. STCs have been described in human, but its physiological role remains unclear. Nowadays, it has been identified the genes that codify for the human homologues of fish STCs, but contrary to fish STC expression, in mammals is much less localized, and as result, several tissues have been reported as possible sources for mammal STCs 2, 3. Nowadays, it has been established that STC1 gen is located within the short arm of the chromosome 8, while STC2 gen was identified at the very end of the long arm of chromosome 5. This different genetic profile, together with the low structural similarity described for both STCs, leads scientists to assume that both proteins may have completely different functions. In fact, concerning their structures, while STC2 conserves around 10 histidine residues in the C‐terminus domain that allows metallic ions binding, STC1 seems to lack this domain 4. Post‐translational modifications of both proteins also differ; thus, STC1 was described to be intracellularly phosphorylated by PKC, which regulates its function, but STC2 is targeted by the ectokinase, casein kinase 2 (CK‐2), in a not well‐defined process that might leads to STC2 secretion 5. Regarding it function, STC1 regulates mineral metabolism and intestinal calcium uptake in mammals, in a similar function to the one described in fish 6. However, there is scarce information about the STC2 physiology, but some authors have proposed that STC2 might regulate intracellular calcium homoeostasis 7, and also it would act as protective protein against reticular stress 8, 9. In this sense, the STC expression has been reported to be altered in severe pathologies, such as oesophageal squamous cancer, breast cancer and thyroid cancer among other types 10, 11; therefore, to improve our knowledge regarding the physiology of these proteins might help to tackle those illness.

On the other hand, STC expression was confirmed in the pancreas tissue; in fact, STC1 was described to be expressed together with insulin in the beta cells of the pancreas islets 12. Furthermore, exogenous STC2 expression in mice resulted in a strong positive stain in the pancreas islets, and subsequent immunohistochemical analysis revealed that STC2 colocalize with glucagon in the alpha cells; thus, the authors inferred a possible role of STC2 in glucose homoeostasis 4. Very recently, STC1 was reported to down‐regulate gluconeogenesis in different kidney cells in rat and fish, and then, it would participate in the overall circulating glucose in mammal 13.

Elevated glycaemia defines the diabetes mellitus (DM). Regarding the causes underlying the appearance of diabetes, both physicians and scientists agreed that there are confluence factors such as an unhealthy diet 14, 15, obesity 16, environmental toxicity 17 and other factors related to the lifestyle 18. Glycosylated haemoglobin has risen as the method of election to diagnose DM2, according to WHO, and thus, over 6% of glycosylated haemoglobin would characterize a DM2 patient (World Health Organization, Geneva, 1999. Report Number: WHO/NCD/NCS/99.2.). However, at this stage, when alteration of haemoglobin becomes evident due to protein undergo glycosylation, probably other proteins within the tissues may have already been glycosylated, leading to tissue damage and dysfunction 19. Therefore, early diagnosis and characterization are fundamental for preventing the tissue damage, and the identification of new early genetic and proteomic markers that help shortening the establishment of the appropriate therapy would be relevant for handle this illness. Herein, using a STC2^‐/‐^ mice that present enhanced post‐natal growth, then it can be also consider as an obesity murine model, we present evidence of a role of STC2 in the development of hyperglycaemia associated with a high‐fat food intake.

## Materials and methods

### Materials

Harlan Teklad 2014 and Harlan Teklad 2018 pellets were supplied by ENVIGO^(R)^ (Valencia, Spain), GLUCOCARD^TM^ G+ meter, ARKRAY Factory Inc. (Shiga, Japan). Human insulin (actrapid) was supplied by Novo Nordisk^(R)^ (Bagsværd, Denmark). Mice food was purchased from Harlam (ENVIGO)^®^(Barcelona, Spain). Anti‐STC1 and anti‐STC‐2 antibodies were from Sigma‐Aldrich^(R)^ (Madrid, Spain). Peroxidase‐conjugated affiniPure goat antimouse and peroxidase‐conjugated affiniPure donkey anti‐rabbit IgGs secondary antibodies were supplied by Jackson ImmunoResearch Ltd.. Glucagon ELISA kit and antiglucagon antibody was purchased from R&D systems (Cat. number DGCG0, Abingdon, UK). FITC‐conjugated secondary antibody was from Santa Cruz Biotechnology (Dallas,TX, USA). Reactive of analytic grade was supplied by Sigma‐Aldrich^(R)^.

### Animals

STC2^‐/‐^ mice were kindly provided by Dr. Roger Reddel (Children's research institute, Australia), while C57BL/6J WT mice were purchased from Harlan (ENVIGO)^®^. Both animal groups were maintained and reproduced at 25°C and 12‐hrs light/dark photoperiod in the animal house of the University of Extremadura. Animals were divided in two main groups and were allowed free access to drink and food that consisted on either regular diet [regular pellet (RP); maintenance diet Harlan Teklad 2014] or enriched diet [breeding pellet (BP); breeding diet, Harlan Teklad 2018]; the breeding pellet consisted in a hypercaloric food; which results in an excess of 0,9 kJ/g containing more protein and fat than RP (Table 1).

**Table 1:** Pellet macronutrient composition and caloric content according to Teklad (ENVIGO)

Teklad Global 14%	Macronutient	% Weight	% Calories
	Crude protein	14.3	20
Energy	Fat	4	13
2.9 (12.1) Kcal/g (KJ/g)	Carbohydrate	48	67
	Crude fibre	4.1	
	Neutral detergent fibre	18	
	Ash	4.7	

Teklad Global 18%	Macronutrient	% Weight	% Calories
	Crude protein	18.6	24
Energy	Fat	6.2	18
3.1 (13) Kcal/g (KJ/g)	Carbohydrate	44.2	58
	Crude fibre	3.5	
	Neutral detergent fibre	14.7	
	Ash	5.3	

### DM2 patients and healthy individuals

DM2 patients were selected of the patients that regularly attend to the Manuel Encinas's health centre for routinely control of their diabetic condition; meanwhile, healthy blood samples were provided by the Extremadura County blood donation centre. Similar number of patients, of both genders, was selected, and their age ranges from 33 to 70 years. All DM2 patients were previously diagnosed due to elevated basal glucose and glycosylated haemoglobin over 6% according to WHO definition. These patients are under different hyperglycaemic medication, as result, some of them presented normoglycaemia and normal haemoglobin glycosylation at the moment of the blood extraction, as confirmed by the biochemical analysis. Blood extraction was performed by nurses of the Extremadura County health Service that belong to the above‐mentioned health centres and following the ethical laws and upon informative acceptance of the patients.

### Mice plasma parameters

Glycaemia—Circulating glucose concentration was determined using a glucometer device (GLUCOCARD^TM^ G+ meter, ARKRAY Factory Inc.) following manufacturer's materials and instructions. Blood was collected from anaesthetized animals (using an isoflurane chamber) from vein of the tail regularly at 8:00 PM, and twice a month.

Glucose oral test—It was performed as described by the Zhan P and collaborators 20. Briefly, 2 mg/g bodyweight was intraperitoneal injected to isoflurane‐anaesthetized mice. Animal starvation for 4 hrs previously to glucose injection was enough to reach and determine basal glycaemia. Upon glucose solution injection, mice glycaemia was determined for 2 hrs, and values were determined as described above and represented in mg/ml.

Insulin tolerance test—Following the indications of the National Mouse Metabolic Phenotyping Center (Massachusetts, USA), one solely insulin intraperitoneal injection (0.5 U/kg) was administered to WT and STC2^‐/‐^ mice that have been previously starved for 4 hrs. In order to ascertain the glycaemia, and subsequently, to diagnose a possible insulin resistance syndrome, we draw three small blood samples from the animals during the following one and a half hour.

Circulating Glucagon test—Blood samples were drawn from the retro‐orbital plexus of isoflurane‐anaesthetized WT and STC2^‐/‐^ mice fed with BP, using a heparinized capillary, which was approved by our institution bioethical committee. Previous to blood extraction, animals were starved for 4 hrs as recommended in the protocol. Mice plasma poor platelet samples were obtained by differential centrifugation, and they were used for determining blood glucagon content according to manufacturer's instruction and using the commercial ELISA kit from R&Dsystems^®^. Glucagon values are presented in ng/ml.

### Histological and tissue analysis

Animals were killed under the effect of isoflurane, and several organs (heart, pancreas, liver, brain) were surgically isolated and injected with ice‐cold 4% paraformaldehyde. Upon determination of the wet weight of each organ, they were immediately derived to the Histology Unit of the Department of Animal Medicine of the Faculty of Veterinary Sciences (University of Extremadura), for establishing possible anatomical and histological difference between mice subgroups. Fixed pancreas was dehydrated in an ascending scale of alcohols and subsequently embedded in paraffin. The 5‐micrometre‐thick sections were routinely stained with haematoxylin–eosin for subsequent histological study.

Non‐superimposed micrographs of each sample were made (40× magnification) using a microscope, obtaining about 1 cm^2^ study surface. In each one, the number of islets, size of the islets and size and number of cells (according to the presence of nucleus) were studied. The data obtained were expressed as unit of measure (mm^2^ or μm^2^, depending on the case). These parameters were performed using a microscope (Eclipse Ni, Nikon^®^, Melville, NY, USA), camera (DS‐Ri, Nikon^®^) and an image analysis program Nis‐elements Br 4.30, Nikon^®^.

Immunohistochemistry—Upon deparaffination and rehydration, mice pancreas section was incubated for 30 min at room temperature with blocking solution (5% BSA in Tris buffer saline medium) to avoid non‐specific binding of the primary antiglucagon antibody provided with the ELISA kit. Primary antibody 1:250 was incubated overnight and upon washing two times with fresh TBS, and FITC‐conjugated secondary antibody was added to the sample for an additional hour. A second round of washing with TBS was required previous to observation of the samples under an epifluorescence inverted microscope (Nikon Diaphot T200). FITC fluorescence was observed using the 450 nm excitation wavelength in to avoid interference with the emission spectra, as well as to facilitate possible erythrocyte infiltration that would denote tissue damage (erythrocyte haemoglobin autofluorecence peak at 415 nm). Epifluorescent images were acquired using a cooled digital CCD camera (Hisca CCD C‐6790, Hamamatsu, Japan) and 40× magnification.

### Western blotting

Blood samples drawn from the healthy individuals, and DM2 patients were processed as described elsewhere 21, 22. Poor platelet plasmas and platelets were isolated by differential centrifugation; subsequently, the samples were fixed and lysed using Laemmli's buffer containing the reducing agent dithiotreitol (5% final concentration). Once all patient blood samples were collected, the proteins were isolated by 15% SDS‐page and subsequent Western blotting was performed using monoclonal anti‐STC1 and anti‐STC2 antibodies (incubated for 1 hr at 1:1000 in TBST supplemented with 5% of BSA). Upon incubating with the adequate secondary antibody, the amount of both proteins was detected using a C‐digit device from LICOR^®^. Analysis of the difference between samples was performed using ImageJ free software from NIH. Additionally, reproving of the membranes with anti–actin antibody was carried out as loading control.

### Statistical analysis

Student's *t*‐test was used for confirming statistical differences among the studied groups. Analysis of the variance was subsequently assessed by performing Dunn's and Tukey's post‐test for multiple comparisons. Furthermore, we have additionally performed Pearson's correlation tests for establishing possible links between different biochemical and demographic parameters observed in the human DM2 patients. Data resulting a *P* < 0.05 are considered as significant, and also, only those parameters that presented a correlation statistically significant are presented in the Results section.

## Results

### Altered STC2 expression in DM2 patients

**Figure 1:**
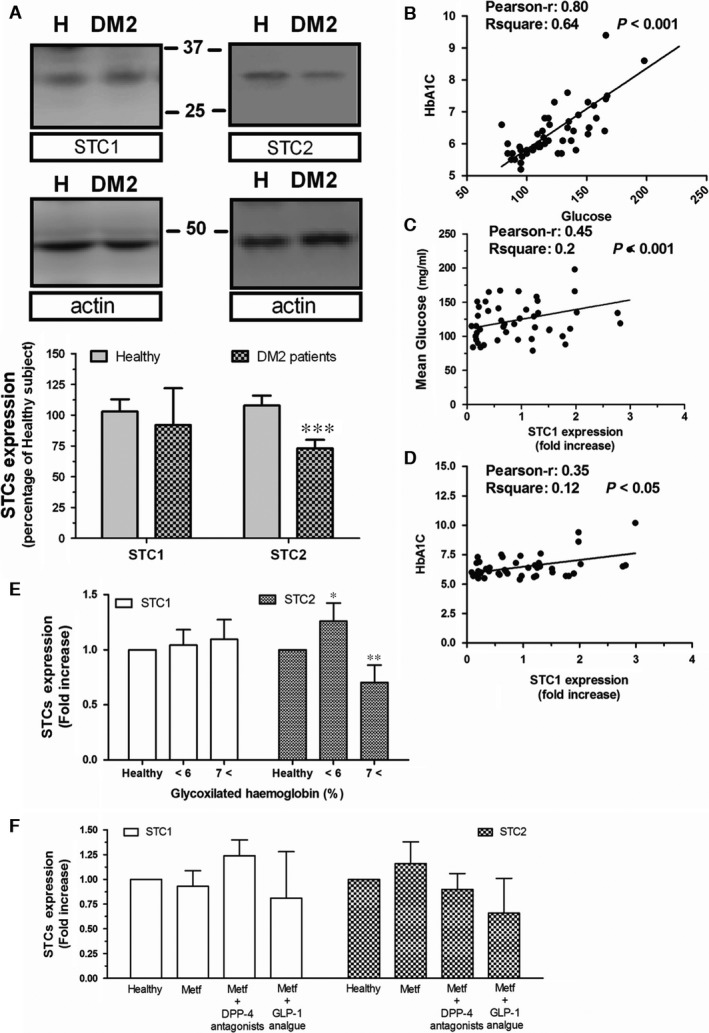
Expression of STCs in platelets drawn from healthy subject and DM2 patients. Platelets isolated from the blood of either healthy individual or DM2 patients were fixed by mixing with Laemmli's buffer under reducing condition (5% final DTT). Subsequent Western blotting was carried out using specific monoclonal anti‐STC1 and STC2 antibodies according to the manufacturer's recommended protocol. Membranes were reprobed with an anti–actin antibody for protein loading control. Bar graphs represent fold increase of STC expression respect STCs found in healthy individuals. Data are representative of the three independent experiments using up to 50 DM2 patients and 12 healthy individuals. (**B–E**) Biochemical variables of DM2 patients were analysed to establish statistical correlation using the Pearson's test. Only those correlations that reached significant values are shown. (**F**) Bar graphs show the regulation of STC expression by different antihyperglycaemic drugs. *, **, ***: represent *P* < 0.05, *P* < 0.01 and *P* < 0.001 as compared to the protein expression found in healthy individuals, as resulted from Student's *t*‐test and Dunn's post‐test for histograms E and F, while Pearson's correlation was used in B‐D.

Human platelets were used to analyse the expression of STCs in human, and platelets have often been used as sentinel cells in the study of certain diseases such as Alzheimer and cancer 23, 24. As demonstrated in Figure 1A, platelets isolated from DM2 patients exhibited a significant reduction of 27.0 ± 7.0% in the STC2 expression, but not in STC1 expression (92.0 ± 30.0%) compared to those from healthy individuals, then revealing a possible relationship between STC2 and glycaemia in human.

By comparing several variables of the DM2 patients, we have confirmed the existence of a very positive correlation between circulating glucose concentration and glycosylated haemoglobin in these patients, in agreement with previous publications (Fig. 1B). In addition, a positive correlation was found between STC1 and glucose concentration, as well as between STC1 and glycosylated haemoglobin (See Fig. 1C and D, respectively), but regarding the latest, no significant differences could be stablished as compared to healthy values (Fig. 1E). Contrary, as previously demonstrated by Moore (Emma E. Moore, Gary Rosenberg, Angela Thostrud, David S. Weigle, Hong Ping Renas, results patented under the Reference number: WWO2001008697 A2), our results showed no direct correlation between STC2 and glycaemia in DM2 patients (data not shown). However, when patients were grouped according to the glycosylated haemoglobin values, we find out that in the DM2 patients who presented the highest glycosylated haemoglobin values (HbAc1 over 7), also presented the lower STC2 expression as compared with the ones found in healthy individuals (Fig. 1E). Interestingly, in the DM2 population analysed, the antiglycaemic medication was unable to significantly modify the expression rate of both STCs; however, the lowest expression of STC2 was found in patients medicated with analogues of GLP‐1, which might be indicative of an alteration in the glucagon mechanism (Fig. 1F).

### Glycaemia in WT and STC2^‐/‐^ mice

**Figure 2:**
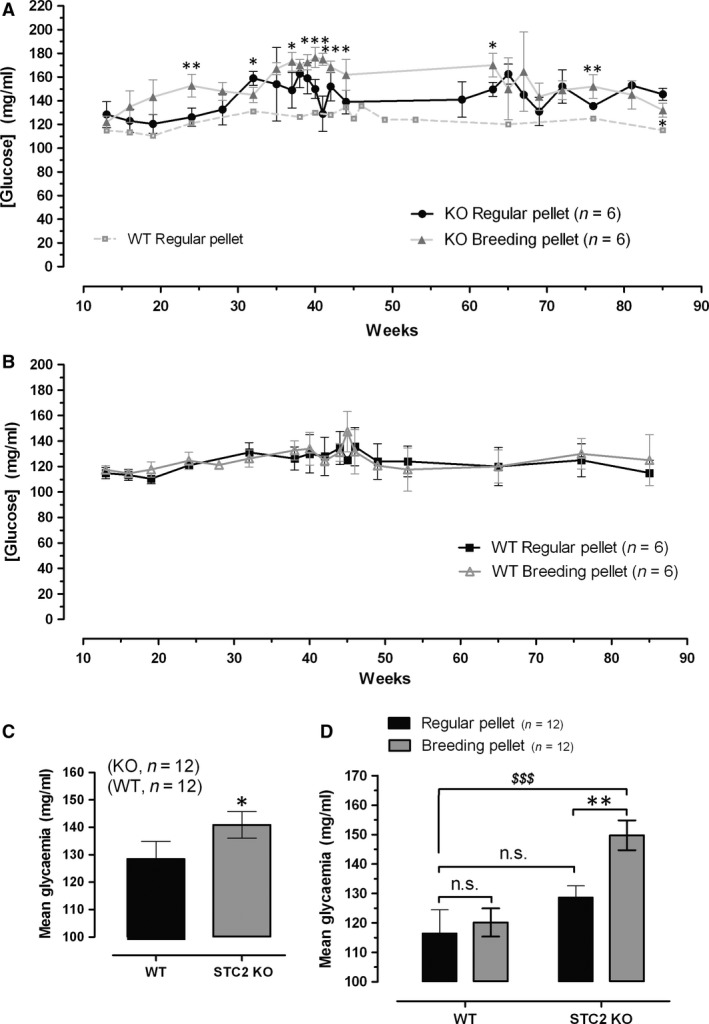
General glycaemia in WT and STC2^‐/‐^ mice according to the type of diet. (**A–B**) Graphs represent the glycaemia of C57BL/6 WT (12 animals) and STC2^‐/‐^ mice (12 animals) analysed according to the type of diet, either regular pellet (Teklad global 2014) or breeding pellet (Teklad global 2018). (**C**) Bar graphs represent the mean glucose concentration during their entire life. (**D**) Bar graphs represent mean glucose concentration among the type of diet used; n.s.: non‐significant. *,**,***represent a *P* < 0.05, *P* < 0.01 and *P* < 0.001, as compared to animals fed with the regular pellet diet; meanwhile, ^*$$$*^represents that *P* < 0.001 as compared to glucose values found in WT fed with Regular pellet. Statistical significance was assessed using Student's *t*‐test (**A–C**) and Tukey's post‐test in **A, B** and **D**.

To ascertain whether STC2 is involved in the regulation of circulating glucose, we have selected C57BL/6 mice both WT and STC2^‐/‐^. This mice strain has been used in different studies related to glucose metabolisms and could be a suitable animal model of diabetes mellitus 25. Thus, preliminary analysis of general glycaemia from WT and STC2^‐/‐^ mice fed with regular pellet (RP) did not report statistical difference among groups (Fig. 2A and B black traces and see also the histogram D). Previous manuscripts reported an elevated predisposition within the obese population for suffering DM2 26, 27, 28, 29, 30, 31, which agree with the fact that most of the patients selected for this study were also overweight (50% of analysed patients have a BMI over 30), and in addition, STC2^‐/‐^ mice are oversize and overweight 32, as it was corroborated in our colonies (reporting a weight of 20.0 ± 1.9 gr and 26.7 ± 1.72 gr in WT and STC2^‐/‐^, respectively, at the 10 weeks of age; *n* = 15–20; meanwhile at the age of 40 weeks, no differences in weight were observed). Hence, we suggested whether STC2^‐/‐^ mice would be a good model for describing the molecular events contributing with the appearance of DM2 in obese patients. Furthermore, animals of both groups were fed with either regular pellet [RP, 2014 Teklad global 14% protein rodent maintenance diets from Harlan (ENVIGO)] or breeding pellet (BP, 2018 Teklad global 18% protein rodent diet). Mice were maintained under normal photoperiod and free access to food and drink. Animals were anaesthetized and blood samples were collected from the vein of the tail twice a month, as described under Materials and Methods Section. As demonstrated in Figure 2A (grey trace), STC2^‐/‐^ mice exhibited a significant higher concentration of blood glucose (these mice presented a mean glucose of 149.7 ± 5.0 mg/ml when fed with BP *versus* 128.6 ± 3.9 mg/ml when are fed with RP. *P* < 0.05; See histogram D in Fig. 2), which is particularly evident until the age of 65 weeks. Upon 65 months, STC2^‐/‐^ feed with both type of food presented similar glycaemia, perhaps due to the genetic predisposition of these mice strains that are often used for obesity and diabetes mellitus murine model. These results would explain the fact that a statistical difference in the general glycaemia is observed between WT and STC2^‐/‐^ mice if data are analysed without considering the type of food use for feeding them (Fig. 2, Histogram C). According to these results, STC2^‐/‐^ mice would represent a good experimental model of hyperglycaemia induced by a hypercaloric diet, and then it could be used as experimental model for establishing possible links between obesity and DM2 appearance.

As shown in the Fig. S1, hyperglycaemic condition of the STC2^‐/‐^ mice fed with BP was further corroborated by performing the oral glucose test; however, this strain does not present insulin resistance according to insulin tolerance test that is also presented in the Fig. S1B. So, hypercaloric diet would evoke pancreatic tissue alteration mainly in STC2^‐/‐^ mice that lead to deregulated insulin production but not insulin action in the tissues.

### Histological alterations in the STC2^‐/‐^ mice pancreas

**Figure 3:**
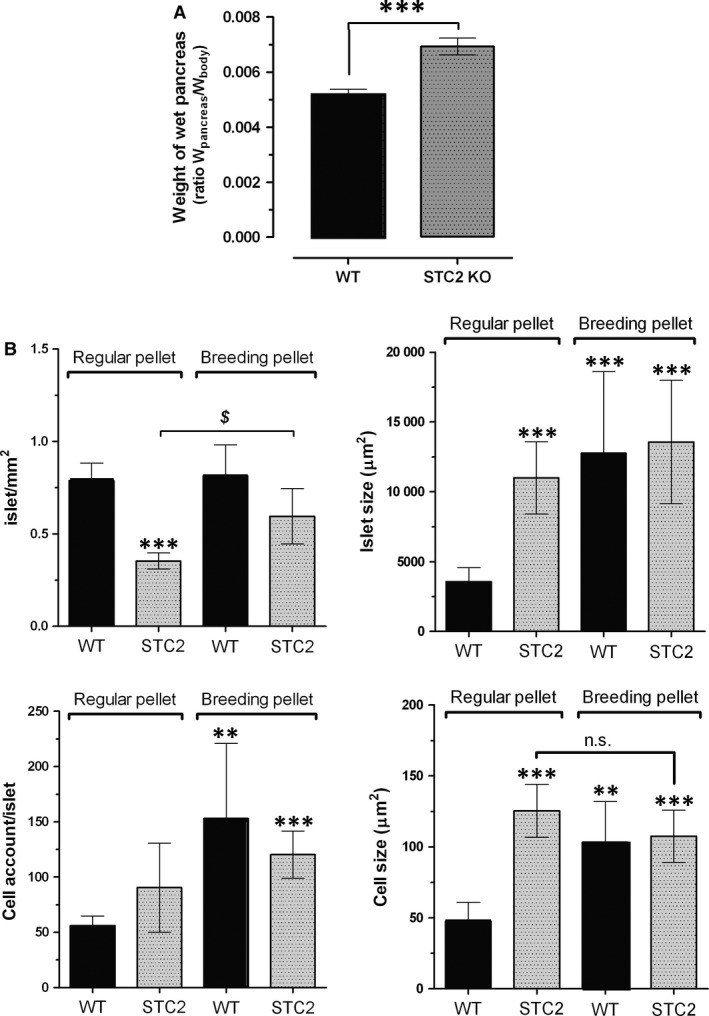
Pancreas size and morphologic changes in the pancreas of WT and STC2^‐/‐^ mice according to the type of diet. WT and STC2^‐/‐^ mice were fed with regular (RP) or hypercaloric breeding diets (BP), and at the age of 40 week, animals were killed (*n* = 4–6 of each group), and subsequent surgical proceed was performed for extracting the pancreas. Pancreas was immediately fixed in 4% paraformaldehyde, after which, wet pancreas weight was estimated using an analytical balance digital precision electronic scale. (**A**) Bar graphs represent either general ratio of the wet pancreas weight respect to the bodyweight. ***represents *P* < 0.001 respect to the ratio of pancreas/bodyweight in WT mice. (**B**) Histological fixation and staining was performed as described under Materials and Methods, and subsequent histological and cellular analysis was performed using the software Nis‐elements Br 4.30, Nikon^®^. **, ***represent *P* < 0.01 and *P* < 0.001, as compared to the values found in WT mice feed with regular pellet (Teklad global 2014). ^*$*^: *P* < 0.05 as compared to the values found in the STC2^‐/‐^ mice fed with regular pellet; Student's *t*‐test (**A**) and Dunn's and Tukey's test was used (**B**).

To delve in the possible alteration evoked by a hypercaloric diet, mice within the range of 40 weeks of age were selected for performing the histological study. Upon determination of their bodyweight, animals were killed as described in the Materials and Methods. Isolated pancreas was weighed and fixed in ice‐cold paraformaldehyde solution (4% w/v). As observed in Figure 3A, the pancreas/bodyweight ratio of the pancreas of STC2^‐/‐^ mice was greater than that from WT mice (0.0069 ± 0.0003 ratio in STC2^‐/‐^ mice *versus* 0.0052 ± 0.0003 ratio in WT mice; *n* = 6, *P* < 0.001).

The histological analysis revealed that STC2^‐/‐^ mice fed with the RP diet presented lesser number of pancreatic islets but of a greater size than WT mice (Fig. 3B), which is consistent with the observation that the pancreatic islets of STC2^‐/‐^ mice presented a slightly higher number of cells (non‐significant), and of bigger size than the cells of WT mice (See Fig. 3B, bottom right‐hand graph), which might explain how STC2^‐/‐^ mice under RP diet can compensate the blood glucose concentration (see Fig. 2). Interestingly, WT mice react to hypercaloric diet (BP diet) by increasing the size of the islets, which is likely due to the greater number of cells that are also bigger in size than in mice feed with RP (See Fig. 3B, black bars in the top right‐hand graph and black bars in the bottom graphs). In contrast, STC2^‐/‐^ mice subjected to hypercaloric BP diet were unable of reorganizing the pancreas tissue with the same efficiency than WT mice did in response to hypercaloric BP diet (See Fig. 3B grey‐dotted bars). Although the number of the pancreas islets increased significantly (Fig. 3B, grey‐dotted bars in the top left‐hand graph), the islet size was not modify in response to BP diet (Fig. 3, grey‐dotted bars in top‐rich hand graph); furthermore, neither changes in the number of cells/islet nor cell size in response to BP was observed in the STC2^‐/‐^ mice (See Fig. 3B bottom graphs), which might explain that STC2^‐/‐^ mice were unable to keep the glycaemia under control as commented above and it is described in the Figure 2.

**Figure 4:**
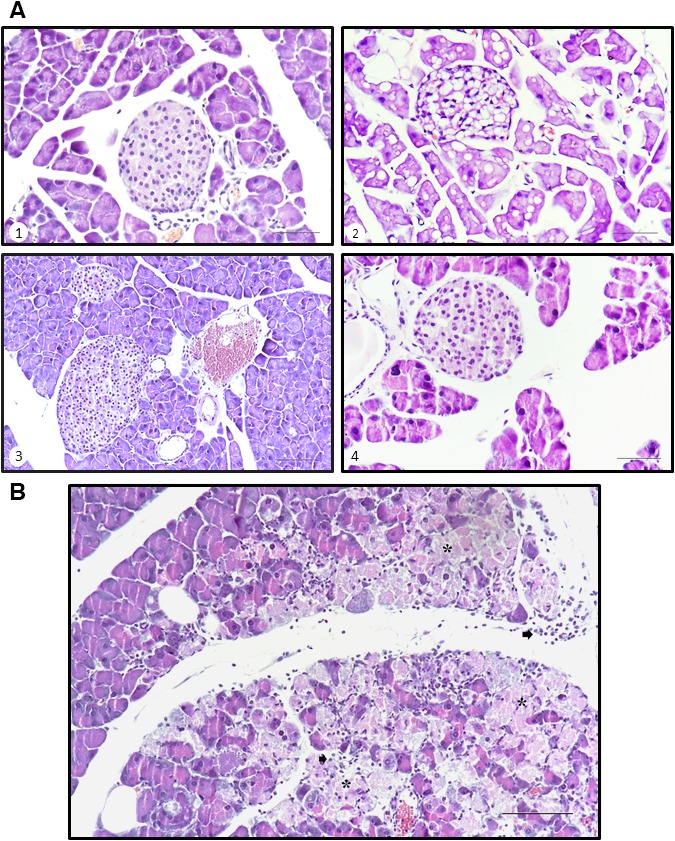
Histological images of Langerhans islets and exocrine pancreas of the study mice. Histological sections were obtained and observed as described in Materials and Methods section. (**A**) WT, regular diet (1), WT, hypercaloric diet (2), KO, regular diet (3), KO, hypercaloric diet (4) (Scale bar=50 μm). (**B**) KO, hypercaloric diet. It is shown necrotic phenomena (*) including inflammatory cells (arrows) (Scale bar=100 μm).

As demonstrated in Figure 4, the histological study revealed cytological differences in the islet components of each study group feed with both types of diets, although more evident in individuals undergoing BP. The islet cells in the WT mice showed a cytoplasm occupied by large vacuoles which might consist of intracellular oil deposit as result of hypercaloric diet as previously reported by others 33, sometimes a single vacuole that displaces the nucleus (compares the different images shown in the Fig. 4A). In contrast, KO individuals showed cells with small and larger vacuoles, giving the cytoplasm a foamy appearance (Fig. 4A‐image 4). It is noteworthy that in the exocrine portion in some KO animals subjected to BP diet, there was evidence of non‐purulent pancreatitis, accompanied in some cases of cellular necrosis (see asterisks in Fig. 4B). Finally, no nuclear changes were observed in any group.

**Figure 5:**
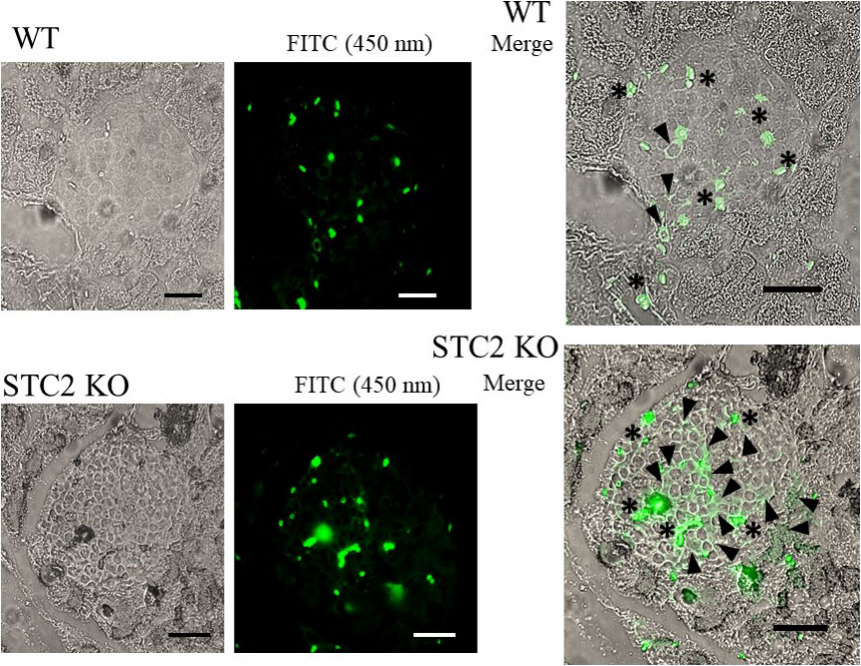
Glucagon immunostaining in WT and STC2^‐/‐^ mice. Pancreatic samples were obtained as described in the Materials and Methods section, upon which non‐specific binding site were blocked. Incubation with the primary antiglucagon antibody (1:250 in TBS) was performed overnight at 4°C. Upon washing with TBS, adequate FITC‐conjugated secondary antibody was used for 30 min. at room temperature; after which, the samples were observed under an inverter epifluorescence microscope. Images were acquired using an excitation wavelength of 450 nm, to avoid excitation and emission overlapping, and are representative of eight to 10 mice preparations. Arrowheads represent positive glucagon staining, then alpha cells or secreted glucagon; meanwhile, asterisk indicates erythrocyte autofluorescence (Scale bar = 50 μm).

In addition, a very positive STC2 expression has been previously describe within the alpha‐islet pancreatic cells; thus, authors inferred a possible glucagon regulation 4. Here, using a primary antiglucagon antibody, and subsequently, a FITC‐conjugated specific secondary antibody, we have observed a more evident positive stain in the pancreas sections of STC2^‐/‐^ mice fed with BP, respect to those sections obtained from WT mice fed under the same regimes (see arrowheads in Fig. 5). Interestingly, pancreatic tissue alteration was also demonstrated by comparing the erythrocyte infiltration observed in the STC2^‐/‐^ slices respect to the WT ones (see asterisks in Fig. 5, that point to haemoglobin autofluorescence).

**Figure 6:**
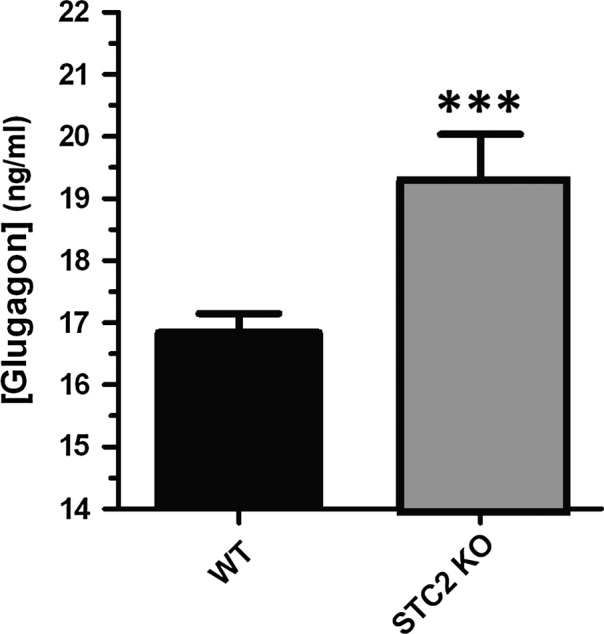
Circulating glucagon in WT and STC2^‐/‐^ mice feed with BP. Blood sample were drawn as described in Materials and Methods section. Upon plasma isolation, it was loaded in a 98‐well ELISA test against glucagon. Histogram represents the average of two independent experiments using STC2 KO (4) and WT (6) fed with BP. ***represents *P* < 0.05 according to the Student's *t*‐test.

Finally, glucagon altered production was also confirmed in Figure 6, where using a commercial ELISA test against glucagon, we were able to detect enhanced circulating glucagon levels in the STC2^‐/‐^ mice fed with BP. Thus, hyperglycaemic condition due to the lack of STC2 expression results from a combination of deregulating insulin and glucagon secretion.

## Discussion

DM2 is a global pandemic disease, affecting up to 415 million of patients according to the international diabetes foundation (www.IDF). Although there are no experimental data available yet, scientists and physicians agree with the fact that the lifestyle adopted by Western societies might contribute to worsening the genetic predisposition of suffering this disease 26, 34. The nowadays lifestyle is characterized by an unhealthy diet combined with an elevated rates of sedentary lifestyle and long working periods full of stressful situations 35, 36. Regarding the latest, in the literature few candidate genes have been pointed for predisposing to suffer DM2 37, 38, 39, 40, and those reported are mainly linked with the glucose and insulin metabolism. Besides to the prevention campaigns, mainly consisting in changing the lifestyle and diet, an early diagnosis would be relevant for tackling the possible collateral damages evoked by high circulating glucose 41. In the present study, some of these previous observations were reproduced in the way that a large number of DM2 included in the present study are also obese, which is worsening the regulation of glycaemia as reflected by the fact that most of them presented elevated glycosylated haemoglobin, despite being under antiglycaemic medication (some of them even required the prescription of two different types of antiglycaemic drugs).

In addition, STCs have emerged as possible markers of diabetes appearance and progression, as it was initially described that STC2 colocalized with glucagon in the alpha cells of the pancreas islets 4 and, later on, these authors demonstrated that contrary to healthy individuals, no correlation was found between circulating insulin and STC2 concentrations in DM2 patients 42. Using an experimental chemical pancreas injury model (consisting in the administration of cerulein), it has been reported that STC2 is overexpressed and might be acting as a protective mechanism 8. In addition, STC1 was proposed to contribute in the regulation of glucose in both rats and fish by affecting to the kidney gluconeogenesis pathway 13; meanwhile, the same research group has also proposed that STC2 would impair glucose oxidation in the liver and muscle (Gonçalves AS, Rossetti CL, Fontella L, Severo L, Kucharski LC, Shein V, Da Silva RSM personal communication). Furthermore, these authors have also proposed that STC2 might regulate the glycogen synthesis from alanine in the liver (Rossetti CL, Master dissertation). However, more experimental evidence is needed for establishing STCs as solid markers of diabetes mellitus. In the present study, we have used the C57BL/6J mice linage, as previously done by others in similar glucose tolerance investigations. These previous studies described that male mice of this linage were able to keep the glucose under strict regulation independently of the age, as demonstrated by performing glucose tolerance test using both oral and intraperitoneal pathways for administering glucose 25. In the present, we reproduced this observation as no significant changes are observed between WT C57BL/6J mice fed either with regular pellet (Teklad 2014) or breeding pellet (Teklad 2018). Furthermore, these authors demonstrated an increased insulin production by the pancreas in response to glucose overload and showed a possible organ adaptive response by evoking pancreas islet enlargement 25. A similar pancreas plasticity was also demonstrated in rats 43. Similarly, under our experimental conditions, WT mice were adequately adapted to hypercaloric diet by evoking the enlargement of the pancreas tissue, but particularly by increasing the size of the pancreas islets and showing cells filled of big vacuoles which consist of oil deposit as previously described 33. Contrary, previous manuscripts reported pancreatic damage derived from high‐fat diet administration which results in fat accumulation in the pancreas and other organs. In other hand, hypercaloric diet also evoked big micro‐ and macrolipid deposits 25, but also we observed islet cell hyperplasia in response to the hypercaloric diet, as confirmed by the fact that the pancreas islets presented more cells of bigger size. In addition, it has been previously observed by others that β‐cells are capable of proliferate under high insulin demanding conditions, due to activation of the endoplasmic reticulum stress adaptive mechanism known as unfolding protein response (UPR) 25, 44. In this sense, STC2 was able to protect the pancreas tissue from the reticular stress evoked by cerulein administration; it is noteworthy that STC2 has been reported as part of the UPR in response to oxidative stress and hypoxia in pancreatic tissue and neurones 8, 9, downstream of PERK, while enhanced expression of STC1 was reported only in response to oxidative stress in tissues like lung and kidneys 45, 46.

On the other hand, STC2^‐/‐^ mice fed with the hypercaloric diet were unable to regulate blood glycaemia. Contrary to what it would be expected, STC2^‐/‐^ mice fed with regular pellet (Teklad 2014) presented a similar glycaemia than WT mice. A possible explanation for this discrepancy relies in the fact that STC2^‐/‐^ mice presented less number of islet in the pancreas but of significant bigger size and contain greater cells, without qualitative differences with the others fed with regular diet. In addition, inflammatory areas and necrotic events have been shown due to stress induced by the diet 47. Then, perhaps these tissue morphologic alterations evoke that STC2^‐/‐^ mice are capable of compensating insulin production under a regular diet, but the pancreatic alterations resulted insufficient for protecting against the higher insulin demanding and stressing situation evoked by a hypercaloric diet. In fact, we have observed altered glucose oral test but not insulin resistance in STC2^‐/‐^ fed with BP, but not in WT mice fed with the same regime. Similarly, STC2 silencing resulted in an elevated intrapancreatic stain of glucagon, and in an enhanced circulating glucagon concentration. As mention above UPR would be in the background of the adaptation mechanism to highly demanding diets, and it has been reported that STC2 would allow cells to survive during UPR 25, 44, 48, then facilitating cell overcome a stressful environmental conditions and to survive to deadly signalling, and even promoting cell proliferation such as occurs in certain types of cancer 49, 50. The latest would explain why cells belonging to pancreas islet of WT mice are able to proliferate under the stressing stimulus of a hypercaloric diet, while STC2^‐/‐^ mice are not capable of adapting to this demanding situation, and subsequently, inflammatory and necrotic phenomena are frequent 47. However, other possible explanation of the enhanced glucagon secretion was observed in the STC2^‐/‐^ mice relays in the negative regulatory function of STC2 over the calcium entry mechanism shown by Zeiger *et al*. 7; hence, lack of STC2 might lead to excessive calcium concentration in the alpha‐islet cells, and subsequently, in a bigger glucagon processing and secretion, so enhancing post‐prandial glycaemia. The latest might explain that patients who required GLP‐1 analogues presented the lowest levels of STC2, although statistical significance could not be stablished with in the analysed population.

The results presented here, together with the fact that STC2^‐/‐^ mice present neonatal and post‐natal oversize 32, lead us to conclude that an alteration in STC2 expression may predispose to suffer DM2 within the obese population. Summarizing, our result would indicate that STCs and particularly STC2 are a suitable marker of DM2 appearance and progression, and might be a linking protein between obesity and DM2.

## Conflict of interest

Authors state that there is no conflict of interest.

## Supporting information


**Figure S1**. GOTT and ITT in WT and STC2^‐/‐^ mice fed with BP for 4 months. GOTT (A) and ITT (B) were performed to WT and STC2‐/‐ mice fed for 4 month with BP and starved for 4 hrs previous to the injection of either glucose solution (2 mg/ml) or insulin (0.5 U.I./Kg). Upon the intraperitoneal injection circulating glucose was tested during the following 120 or 90 hrs respectively as described in material and methods section. Data result of the average of four STC2 KO and six WT mice. *represents *P* < 0.05 according to Student's *t*‐test.

## References

[1] Chang AC , Reddel RR . Identification of a second stanniocalcin cDNA in mouse and human: stanniocalcin 2. Mol Cell Endocrinol. 1998; 141: 95–9. 9723890 10.1016/s0303-7207(98)00097-5

[2] Yeung BH , Law AY , Wong CK . Evolution and roles of stanniocalcin. Mol Cell Endocrinol. 2012; 349: 272–80. 22115958 10.1016/j.mce.2011.11.007

[3] Ishibashi K , Imai M . Prospect of a stanniocalcin endocrine/paracrine system in mammals. Am J Physiol Renal Physiol. 2002; 282: F367–75. 11832417 10.1152/ajprenal.00364.2000

[4] Moore EE , Kuestner RE , Conklin DC , *et al* Stanniocalcin 2: characterization of the protein and its localization to human pancreatic alpha cells. Horm Metab Res. 1999; 31: 406–14. 10450831 10.1055/s-2007-978764

[5] Jellinek DA , Chang AC , Larsen MR , *et al* Stanniocalcin 1 and 2 are secreted as phosphoproteins from human fibrosarcoma cells. Biochem J. 2000; 350(): 453–61. 10947959 PMC1221272

[6] Xiang J , Guo R , Wan C , *et al* Regulation of Intestinal Epithelial Calcium Transport Proteins by Stanniocalcin‐1 in Caco2 Cells. Int J Mol Sci. 2016; 17: pii:E1095. 27409607 10.3390/ijms17071095 PMC4964471

[7] Zeiger W , Ito D , Swetlik C , *et al* Stanniocalcin 2 is a negative modulator of store‐operated calcium entry. Mol Cell Biol. 2011; 31: 3710–22. 21746875 10.1128/MCB.05140-11 PMC3165734

[8] Fazio EN , Dimattia GE , Chadi SA , *et al* Stanniocalcin 2 alters PERK signalling and reduces cellular injury during cerulein induced pancreatitis in mice. BMC Cell Biol. 2011; 12: 17–28. 21545732 10.1186/1471-2121-12-17 PMC3224136

[9] Law AY , Wong CK . Stanniocalcin‐2 is a HIF‐1 target gene that promotes cell proliferation in hypoxia. Exp Cell Res. 2010; 316: 466–76. 19786016 10.1016/j.yexcr.2009.09.018

[10] Jeon M , Han J , Nam SJ , *et al* STC‐1 expression is upregulated through an Akt/NF‐kappaB‐dependent pathway in triple‐negative breast cancer cells. Oncol Rep. 2016; 36: 1717–22. 27461417 10.3892/or.2016.4972

[11] Hou J , Wang Z , Xu H , *et al* Stanniocalicin 2 suppresses breast cancer cell migration and invasion *via* the PKC/claudin‐1‐mediated signaling. PLoS ONE. 2015; 10: e0122179. 25830567 10.1371/journal.pone.0122179 PMC4382185

[12] Zaidi D , Turner JK , Durst MA , *et al* Stanniocalcin‐1 co‐localizes with insulin in the pancreatic islets. ISRN Endocrinol. 2012; 2012: 834359. 23119181 10.5402/2012/834359 PMC3479999

[13] Schein V , Kucharski LC , Guerreiro PM , *et al* Stanniocalcin 1 effects on the renal gluconeogenesis pathway in rat and fish. Mol Cell Endocrinol. 2015; 414: 1–8. 26187698 10.1016/j.mce.2015.07.010

[14] Bahadoran Z , Mirmiran P , Azizi F . Fast Food Pattern and Cardiometabolic Disorders: a Review of Current Studies. Health Promot Perspect. 2015; 5: 231–40. 26933642 10.15171/hpp.2015.028 PMC4772793

[15] Ley SH , Hamdy O , Mohan V , *et al* Prevention and management of type 2 diabetes: dietary components and nutritional strategies. Lancet. 2014; 383: 1999–2007. 24910231 10.1016/S0140-6736(14)60613-9 PMC4751088

[16] Orio F , Muscogiuri G , Nese C , *et al* Obesity, type 2 diabetes mellitus and cardiovascular disease risk: an uptodate in the management of polycystic ovary syndrome. Eur J Obstet Gynecol Reprod Biol. 2016; 207: 214–219. 27575870 10.1016/j.ejogrb.2016.08.026

[17] Schumacher L , Abbott LC . Effects of methyl mercury exposure on pancreatic beta cell development and function. J Appl Toxicol. 2016; 37(1): 4–12. 27594070 10.1002/jat.3381

[18] Reis JP , Loria CM , Sorlie PD , *et al* Lifestyle factors and risk for new‐onset diabetes: a population‐based cohort study. Ann Intern Med. 2011; 155: 292–9. 21893622 10.1059/0003-4819-155-5-201109060-00006 PMC3491359

[19] Preciado‐Puga MC , Malacara JM , Fajardo‐Araujo ME , *et al* Markers of the progression of complications in patients with type 2 diabetes: a one‐year longitudinal study. Exp Clin Endocrinol Diabetes. 2014; 122: 484–90. 25230243 10.1055/s-0034-1372594

[20] Zhang P . Glucose Tolerance Test in Mice. Bio‐protocol Bio. 2011; 101: e159 DOI: 10.21769/BioProtoc.159

[21] Rosado JA , Graves D , Sage SO . Tyrosine kinases activate store‐mediated Ca2 + entry in human platelets through the reorganization of the actin cytoskeleton. Biochem J. 2000; 351(): 429–37. 11023829 PMC1221379

[22] Saavedra FR , Redondo PC , Hernandez‐Cruz JM , *et al* Store‐operated Ca(2 +) entry and tyrosine kinase pp60(src) hyperactivity are modulated by hyperglycemia in platelets from patients with non insulin‐dependent diabetes mellitus. Arch Biochem Biophys. 2004; 432: 261–8. 15542065 10.1016/j.abb.2004.09.034

[23] Zainaghi IA , Talib LL , Diniz BS , *et al* Reduced platelet amyloid precursor protein ratio (APP ratio) predicts conversion from mild cognitive impairment to Alzheimer's disease. J Neural Transm (Vienna). 2012; 119: 815–9. 22573143 10.1007/s00702-012-0807-x

[24] Hu Q , Wang M , Cho MS , *et al* Lipid profile of platelets and platelet‐derived microparticles in ovarian cancer. BBA Clin. 2016; 6: 76–81. 27453821 10.1016/j.bbacli.2016.06.003 PMC4941562

[25] Leiter EH , Premdas F , Harrison DE , *et al* Aging and glucose homeostasis in C57BL/6J male mice. Faseb J. 1988; 2: 2807–11. 3044905 10.1096/fasebj.2.12.3044905

[26] Miranda‐Massari JR , Gonzalez MJ , Fernando AS , *et al* Metabolic Correction as a tool to improve diabetes type 2 management. Bol Asoc Med P R. 2015; 107: 54–9. 26434085

[27] Guijarro De Armas MG , Monereo Megias S , Civantos Modino S , *et al* Prevalence of carbohydrate metabolism disturbances in a population of children and adolescents with severe obesity. Endocrinol Nutr. 2010; 57: 467–71. 21051299 10.1016/j.endonu.2010.09.002

[28] Xekouki P , Nikolakopoulou NM , Papageorgiou A , *et al* Glucose dysregulation in obese children: predictive, risk, and potential protective factors. Obesity (Silver Spring). 2007; 15: 860–9. 17426321 10.1038/oby.2007.600

[29] Molist‐Brunet N , Jimeno‐Mollet J . Franch‐Nadal J [Correlation between the various measurements of obesity and the degree of resistance to insulin]. Aten Primaria. 2006; 37: 30–6. 16545301 10.1157/13083939 PMC8149154

[30] Gomes MB , Gianella D , Faria M , *et al* Prevalence of Type 2 diabetic patients within the targets of care guidelines in daily clinical practice: a multi‐center study in Brazil. Rev Diabet Stud. 2006; 3: 82–7. 17487331 10.1900/RDS.2006.3.82 PMC1783582

[31] Dilla T , Costi M , Boye KS . et al [The impact of obesity in the management and evolution of diabetes mellitus]. Rev Clin Esp. 2008; 208: 437–43. 19000471 10.1157/13127604

[32] Chang AC , Hook J , Lemckert FA , *et al* The murine stanniocalcin 2 gene is a negative regulator of postnatal growth. Endocrinology. 2008; 149: 2403–10. 18258678 10.1210/en.2007-1219

[33] Fraulob JC , Ogg‐Diamantino R , Fernandes‐Santos C , *et al* A Mouse Model of Metabolic Syndrome: insulin Resistance, Fatty Liver and Non‐Alcoholic Fatty Pancreas Disease (NAFPD) in C57BL/6 Mice Fed a High Fat Diet. J Clin Biochem Nutr. 2010; 46: 212–23. 20490316 10.3164/jcbn.09-83 PMC2872226

[34] de Leon AC , Rodriguez JC , Coello SD . et al [Lifestyle and treatment adherence of type 2 diabetes mellitus people in the Canary Islands]. Rev Esp Salud Publica. 2009; 83: 567–75. 19893884

[35] Verwey R , van der Weegen S , Spreeuwenberg M , *et al* A monitoring and feedback tool embedded in a counselling protocol to increase physical activity of patients with COPD or type 2 diabetes in primary care: study protocol of a three‐arm cluster randomised controlled trial. BMC Fam Pract. 2014; 15: 93. 24885096 10.1186/1471-2296-15-93 PMC4030038

[36] Verwey R , van der Weegen S , Spreeuwenberg M , *et al* A pilot study of a tool to stimulate physical activity in patients with COPD or type 2 diabetes in primary care. J Telemed Telecare. 2014; 20: 29–34. 24414397 10.1177/1357633X13519057

[37] Artuso R , Provenzano A , Mazzinghi B , *et al* Therapeutic implications of novel mutations of the RFX6 gene associated with early‐onset diabetes. Pharmacogenomics J. 2015; 15: 49–54. 25048417 10.1038/tpj.2014.37

[38] Piccini B , Artuso R , Lenzi L , *et al* Clinical and molecular characterization of a novel INS mutation identified in patients with MODY phenotype. Eur J Med Genet. 2016; 59: 590–595. 27659712 10.1016/j.ejmg.2016.09.016

[39] Kleinberger JW , Maloney KA , Pollin TI . The Genetic Architecture of Diabetes in Pregnancy: implications for Clinical Practice. Am J Perinatol. 2016; 33: 1319–1326. 27571483 10.1055/s-0036-1592078 PMC5507691

[40] Stekelenburg CM , Schwitzgebel VM . Genetic Defects of the beta‐Cell That Cause Diabetes. Endocr Dev. 2016; 31: 179–202. 26824366 10.1159/000439417

[41] Luo M , Li R , Deng X , *et al* Platelet‐derived miR‐103b as a novel biomarker for the early diagnosis of type 2 diabetes. Acta Diabetol. 2015; 52: 943–9. 25820527 10.1007/s00592-015-0733-0

[42] Moore EE , Rosenberg G , Thostrud A , *et al* Use of stanniocalcin 2 in the treatment of type ii diabetes and complications thereof. Google Patents, 2001.

[43] Magal E , Chaudhuri M , Adelman RC . The capability for regulation of insulin secretion by somatostatin in purified pancreatic islet B cells during aging. Mech Ageing Dev. 1986; 33: 139–46. 2870220 10.1016/0047-6374(86)90022-9

[44] Sharma RB , O'Donnell AC , Stamateris RE , *et al* Insulin demand regulates beta cell number *via* the unfolded protein response. J Clin Invest. 2015; 125: 3831–46. 26389675 10.1172/JCI79264 PMC4607122

[45] Tang SE , Wu CP , Wu SY , *et al* Stanniocalcin‐1 ameliorates lipopolysaccharide‐induced pulmonary oxidative stress, inflammation, and apoptosis in mice. Free Radic Biol Med. 2014; 71: 321–31. 24685991 10.1016/j.freeradbiomed.2014.03.034

[46] Huang L , Belousova T , Chen M , *et al* Overexpression of stanniocalcin‐1 inhibits reactive oxygen species and renal ischemia/reperfusion injury in mice. Kidney Int. 2012; 82: 867–77. 22695329 10.1038/ki.2012.223 PMC3443530

[47] Oliveira RB , Maschio DA , Carvalho CP , *et al* Influence of gender and time diet exposure on endocrine pancreas remodeling in response to high fat diet‐induced metabolic disturbances in mice. Ann Anat. 2015; 200: 88–97. 25819502 10.1016/j.aanat.2015.01.007

[48] Ito D , Walker JR , Thompson CS , *et al* Characterization of stanniocalcin 2, a novel target of the mammalian unfolded protein response with cytoprotective properties. Mol Cell Biol. 2004; 24: 9456–69. 15485913 10.1128/MCB.24.21.9456-9469.2004 PMC522226

[49] Wang Y , Gao Y , Cheng H , *et al* Stanniocalcin 2 promotes cell proliferation and cisplatin resistance in cervical cancer. Biochem Biophys Res Commun. 2015; 466: 362–8. 26361149 10.1016/j.bbrc.2015.09.029

[50] Na SS , Aldonza MB , Sung HJ , *et al* Stanniocalcin‐2 (STC2): a potential lung cancer biomarker promotes lung cancer metastasis and progression. Biochim Biophys Acta. 2015; 1854: 668–76. 25463045 10.1016/j.bbapap.2014.11.002

