# Supplementary figures and images for: Involvement of stanniocalcins in the deregulation of glycaemia in obese mice and type 2 diabetic patients
Source: J Cell Mol Med. 2017 Oct 9;22(1):684–94. doi: 10.1111/jcmm.13355 (PMC5742690; doi:10.1111/jcmm.13355)

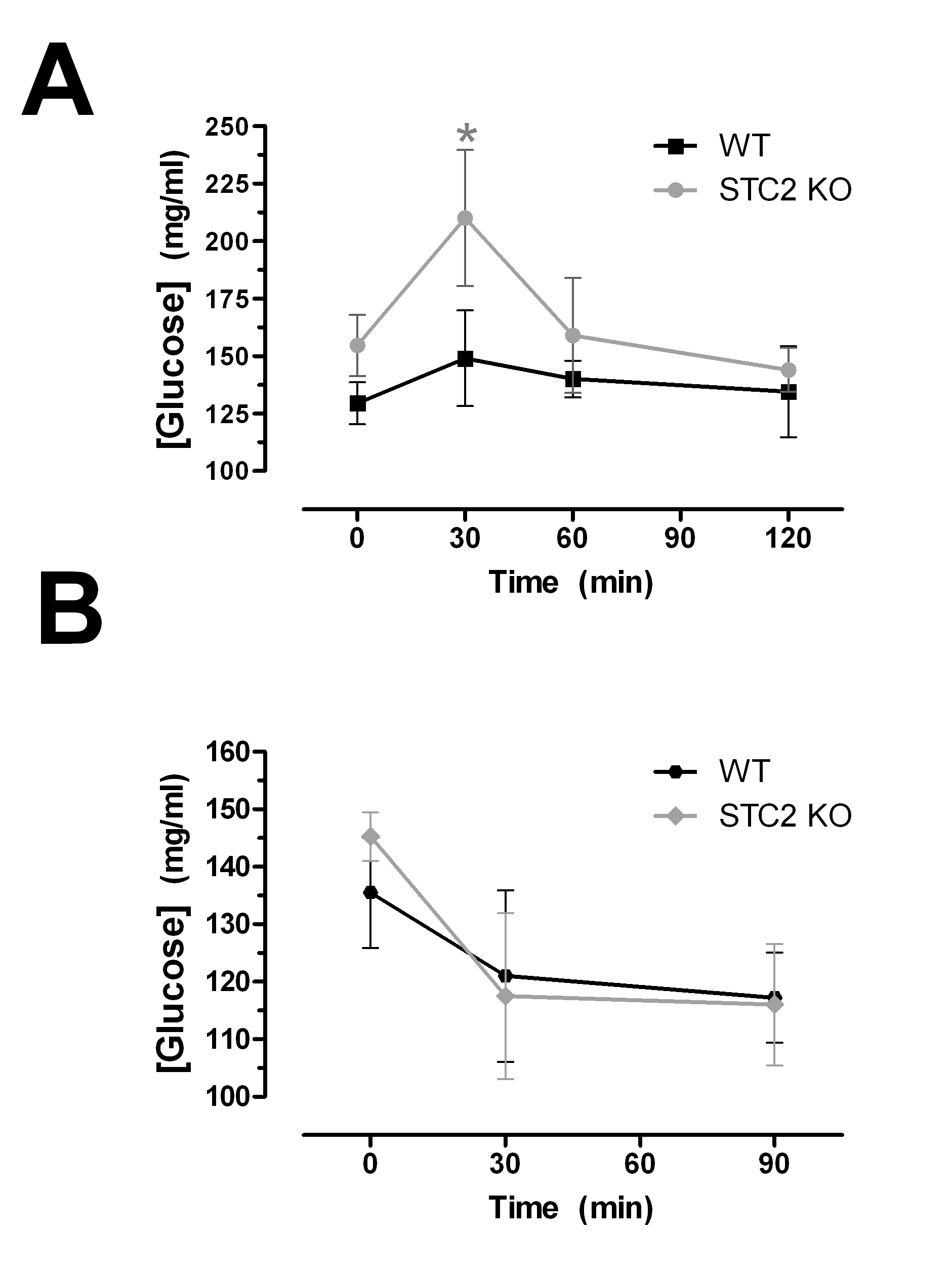

Supplement: Supplementary file 1 — Figure S1. GOTT and ITT in WT and STC2‐/‐ mice fed with BP for 4 months. GOTT (A) and ITT (B) were performed to WT and STC2‐/‐ mice fed for 4 month with BP and starved for 4 hrs previous to the injection of either glucose solution (2 mg/ml) or insulin (0.5 U.I./Kg). Upon the intraperitoneal injection circulating glucose was tested during the following 120 or 90 hrs respectively as described in material and methods section. Data result of the average of four STC2 KO and six WT mice. *represents P < 0.05 according to Student's t‐test. [file JCMM-22-684-s001.tif]
